# Overview of Community-Acquired Pneumonia and the Role of Inflammatory Mechanisms in the Immunopathogenesis of Severe Pneumococcal Disease

**DOI:** 10.1155/2013/490346

**Published:** 2013-12-25

**Authors:** Helen C. Steel, Riana Cockeran, Ronald Anderson, Charles Feldman

**Affiliations:** ^1^Medical Research Council Unit for Inflammation and Immunity, Department of Immunology, Faculty of Health Sciences, University of Pretoria and Tshwane Academic Division of the National Health Laboratory Service, P.O. Box 2034, Pretoria 0001, South Africa; ^2^Division of Pulmonology, Department of Internal Medicine, Charlotte Maxeke Johannesburg Academic Hospital, Faculty of Health Sciences, University of the Witwatersrand, Johannesburg 2193, South Africa

## Abstract

Community-acquired pneumonia (CAP) remains a leading cause of morbidity and mortality among the infectious diseases. Despite the implementation of national pneumococcal polyvalent vaccine-based immunisation strategies targeted at high-risk groups, *Streptococcus pneumoniae* (the pneumococcus) remains the most common cause of CAP. Notwithstanding the HIV pandemic, major challenges confronting the control of CAP include the range of bacterial and viral pathogens causing this condition, the ever-increasing problem of antibiotic resistance worldwide, and increased vulnerability associated with steadily aging populations in developed countries. These and other risk factors, as well as diagnostic strategies, are covered in the first section of this review. Thereafter, the review is focused on the pneumococcus, specifically the major virulence factors of this microbial pathogen and their role in triggering overexuberant inflammatory responses which contribute to the immunopathogenesis of invasive disease. The final section of the review is devoted to a consideration of pharmacological, anti-inflammatory strategies with adjunctive potential in the antimicrobial chemotherapy of CAP. This is focused on macrolides, corticosteroids, and statins with respect to their modes of anti-inflammatory action, current status, and limitations.

## 1. Overview of Community-Acquired Pneumonia

### 1.1. Introduction

Community-acquired pneumonia (CAP) is commonly described as an acute infection of the lung parenchyma acquired in the community. It is most commonly bacterial in nature and is associated with clinical and/or radiological evidence of consolidation of part or parts of one or both lungs [1]. CAP is associated with a considerable burden of disease in most regions of the world [2–6]. It is one of the most important serious infectious diseases, accounting for a considerable number of hospital admissions, with an increasing incidence in many parts of the world and an increasing rate of serious complications [7]. As part of the burden of respiratory infections, CAP is well recognised to be a leading cause of death among the infectious diseases [6, 8]. The reason that CAP is so common relates to the very high prevalence of specific risk factors for this infection in patients worldwide [6]. While a myriad of microorganisms may cause CAP, in reality a relatively small number of pathogens predominate, in particular the bacteria, of which *Streptococcus pneumoniae *(pneumococcus) is by far the most common [7].

There is considerable concern about the emerging resistance among the usual CAP pathogens to the most commonly used antimicrobial agents. There are a number of important decisions that need to be made with regard to the assessment and management of patients with CAP, not least of which is an evaluation of the severity of the infection [6]. A number of guidelines have been published worldwide, describing the optimal treatment of patients with CAP, with the aim of improving patient outcomes.

The remainder of this introductory overview of CAP will focus on (i) burden of disease, (ii) risk factors, (iii) microbiology, (iv) antimicrobial chemotherapy, (v) antibiotic resistance, and (vi) assessment of severity of illness using clinical scoring systems and laboratory biomarkers individually and in combination.

### 1.2. Burden of Disease

CAP continues to be a cause of considerable morbidity and mortality in most parts of the world, being the most frequent infectious cause of death in patients in the USA and throughout Europe [9]. Since aging is a significant risk factor for this infection and given that in many areas of the world, such as Europe, the population is aging, an increase in incidence in the next decades is anticipated [9]. The mechanism by which aging is associated with a risk for CAP is multifactorial, not simply related to chronological age, but frequently associated with (i) underlying comorbid conditions that more commonly occur in the aging population; (ii) a greater risk of being infected with antibiotic-resistant pathogens; (iii) social factors; and (iv) even place of residence [6]. Studies in the USA [2], Europe [5], Latin America [3], and the Asia-Pacific region [4] attest to the fact that CAP has a substantial clinical and economic burden, a high rate of antibiotic resistance among the pathogens, and a significant effect on both immediate and long-term prognosis, as well as on the quality of life of infected patients. Given this high burden of disease, it is recommended that steps be taken to ensure appropriate treatment, ongoing surveillance for antimicrobial resistance among the common pathogens, and strategies, including vaccination, to prevent these infections.

### 1.3. Risk Factors

As shown in the list below, there is a considerable number of risk factors for CAP that exist in populations all over the world, and most of these risk factors are associated with an impairment of the efficacy of host immune defence [7]. Many of these risk factors are also associated with a greater mortality risk [6]. In addition to aging, the common risk factors in adults are smoking, the presence of various underlying comorbid conditions, including chronic cardiorespiratory, renal and hepatic conditions, and, at least in some regions of the world, concomitant human immunodeficiency virus (HIV) infection [7]. There is also some evidence that male patients and those of certain racial or ethnic groups may be at greater risk of pneumonia [6].

Risk factors for community-acquired pneumonia are as follows:

- extremes of age (very young and the aging),
- male gender,
- certain populations (various racial or ethnic groups),
- lifestyle factors (excessive alcohol consumption and smoking),
- underlying comorbid conditions such as
  - chronic cardiorespiratory illnesses,
  - chronic renal disorders,
  - hepatic conditions,
  - diabetes mellitus,
  - neoplastic diseases,
  - human immunodeficiency virus infection,
- medications (e.g., inhaled corticosteroids, proton pump inhibitors),
- additional risk factors associated with pneumococcal infections in particular (e.g., myeloma, hypogammaglobulinemia (such as IgG2 deficiency), surgical asplenia, or “functional” asplenia (such as in sickle cell disease).

Smoking, both active and passive (particularly in children), is a well-described risk factor for CAP, particularly in HIV-infected persons, as well as for many other infectious diseases, and this has been comprehensively reviewed recently [10]. The main mechanisms for this predisposition relate to the suppressive effect that smoking has on the protective actions of the airway mucociliary clearance mechanism, on the various components of the innate and adaptive immune systems of the host, as well as direct effects on microbial pathogens that promote their virulence, and possibly antibiotic resistance [10].

Several comorbid factors relate quite closely to the risk of CAP and the possibility of more severe illness, as well as to the likelihood of a worse outcome [6]. Among the most common comorbid predisposing factors to CAP, as mentioned above, are chronic obstructive pulmonary disease, congestive cardiac failure, diabetes mellitus, a high intake of alcohol, and smoking [6]. However, various other conditions, including those of the neurological system, the liver, the kidney, and also neoplastic disease, represent important risk factors. More recently, there has been considerable interest in the fact that inhaled medication (particularly inhaled corticosteroids) appears to be a risk factor for CAP and this has been reviewed elsewhere [11].

In a number of regions of the world, such as in sub-Saharan Africa, concomitant infection with HIV represents a major risk factor for CAP and has been extensively reviewed elsewhere [12–14]. The spectrum of bacterial pathogens causing CAP in HIV-infected patients is very similar to that in HIV-uninfected patients with *Streptococcus pneumoniae *(pneumococcus) predominating [13]. The clinical presentation of CAP in HIV-infected persons, particularly pneumococcal CAP, is similar to that among HIV-uninfected patients except that the patients are frequently younger, of the female sex, and have a greater frequency of respiratory symptoms [13, 14]. Furthermore, there is a similar spectrum of disease severities, with the commonly used severity of illness scores appearing to have an equivalent value in predicting outcome compared to HIV-uninfected individuals. However, when cases with bacteremic pneumococcal CAP are stratified according to age and severity of illness, HIV-infected persons have been found to have a significantly higher mortality with an increasing trend as the CD4 cell count decreases [14]. Some investigators have therefore recommended that the decision to admit HIV-infected patients with CAP to hospital should be based on both the CD4 cell count and the severity of illness, those with a CD4 cell count <200/*μ*L blood always being admitted to hospital, and those with a higher CD4 cell count being admitted to hospital if warranted according to the severity of illness [12].

Recent studies have identified several mechanisms, specifically the presence of gene polymorphisms, including single nucleotide polymorphisms (SNPs) in genes encoding proteins of the innate immune system, which contribute not only to susceptibility for development of CAP, but also to a worse outcome. For example, SNPs in the IL-6 gene, specifically IL-6 174 G/G, have been reported to protect patients with pneumococcal CAP against development of ARDS, septic shock, and multiple organ dysfunction syndrome, resulting in less severe disease and lower mortality [15]. In addition, investigation of the role of SNPs in the genes encoding the surfactant proteins (SP) A, B, C, and D revealed associations with both susceptibility for both development of CAP and more severe disease [16, 17]. Similar, albeit statistically insignificant, findings were reported for SP-A and SP-D in patients with pneumococcal CAP [17].

### 1.4. Microbiology of CAP

The most prominent organism causing CAP is the pneumococcus, and this remains true irrespective of the severity of infection across the spectrum of outpatients, inpatients not in the intensive care unit (ICU), and even cases with CAP requiring ICU admission [11, 18]. Studies in Europe, the USA, Latin America, the Asia-Pacific region, and elsewhere attest to the fact that the pneumococcus is consistently documented to be the most predominant pathogen [2–5, 9]. Interestingly, while it is well described that pneumococcal infections commonly complicate both seasonal and pandemic influenza infections, more recently it was documented that the pneumococcus was a common bacterial coinfection in patients with influenza A H1N1 infection who were admitted to hospital with CAP [19–21]. In the former two studies, the pneumococcus was the most common bacterial cause of bacterial co-infection, accounting for 62% and 54.8% of cases, respectively, and being associated with a greater risk of septic shock or need for vasopressors, as well as increased need for mechanical ventilation and a longer ICU stay [20, 21].

Many recent studies of pneumococcal infection have focussed on the issue of pneumococcal serotypes causing disease, and the possible association of different serotypes with disease severity, particularly in relation to the release of the newer pneumococcal conjugate vaccines and the extended indication for their use in adults [22–25]. While earlier studies suggested that host factors may be more important than isolate serotype in determining the severity and outcome of pneumococcal infections [22], suggesting that vaccination was unlikely to be associated with a change in these end-points, more recent studies suggest that IPD outcome is a serotype-related issue; for example, serotype 3 is more commonly associated with septic shock [24, 25]. When considering the likely benefit of vaccination, it also remains important to consider the serotypes implicated in nonbacteremic infections, which studies have suggested that due to greater serotype distribution are less comprehensively covered by currently available conjugate vaccines [26, 27].

After the pneumococcus, the next most common pathogens are the so-called atypical pathogens, the respiratory viruses, and *Haemophilus influenzae *[11]. Among the viruses, influenza predominates, but smaller numbers of various other respiratory viruses are also documented [11, 28, 29]. Less commonly, additional pathogens are documented, particularly in cases with respiratory-related comorbid illnesses. These include *Staphylococcus aureus, Pseudomonas aeruginosa*, and the Enterobacteriaceae [11]. The pathogens causing CAP have been extensively reviewed elsewhere [11].

### 1.5. Antimicrobial Chemotherapy of Bacterial CAP

On presentation of adults with suspected CAP, empiric antimicrobial chemotherapy is initiated according to the relevant national guidelines, with age, comorbidities, and disease severity being the primary determinants of the class of antibiotic(s) and route of administration. In the case of outpatient therapy, previously healthy patients who had not received antibiotics during the 3-month period prior to presentation, monotherapy with a macrolide or doxycycline is recommended by the Infectious Diseases Society of America (IDSA)/American Thoracic Society (ATS); in those with comorbidities and/or prior recent use of antibiotics, the recommendation is the combination of an antipneumococcal *β*-lactam and a macrolide, or alternatively, a respiratory fluoroquinolone [30]. In the case of inpatient therapy in the non-ICU setting, the guidelines advocate the combination of a *β*-lactam and a macrolide, or, alternatively, monotherapy with a respiratory fluoroquinolone. In the case of patients admitted to intensive care, the combination of a *β*-lactam with either a macrolide or a respiratory fluoroquinolone is recommended [30].

Clearly, these therapeutic strategies can be reevaluated on the basis of clinical response and acquisition of microbiological and other laboratory data.

#### 1.5.1. Combination Antibiotic Therapy

The rationale for implementation of combination therapy (various combinations, but most frequently a *β*-lactam with a macrolide) in patients with severe CAP was largely based on a series of observational studies, both retrospective and prospective, conducted between 1999 and 2010, which demonstrated significantly lower in-hospital or ICU mortality [31]. Although the microbiological mechanisms underpinning the apparent benefit of combination antibiotic therapy remain unknown, activity of macrolides/fluoroquinolones/tetracyclines against the atypical pathogens *Chlamydia pneumoniae, Legionella pneumophila,* and *Mycoplasma pneumoniae*, as well as efficacy in eradicating polymicrobial sepsis, have been proposed [32]. When considering these possible mechanisms, it is also important to note that the benefit of adding a macrolide to standard *β*-lactam therapy is particularly evident in sicker patients, such as those with severe sepsis due to pneumonia, and in intubated patients [31, 33]. Benefit also extends to cases infected with macrolide-resistant pathogens (e.g., macrolide-resistant pneumococcal infections and even to cases with gram-negative infections) [33]. The aforementioned studies, as well as several others which failed to confirm a survival benefit when comparing monotherapy with combination therapy, have recently been extensively reviewed elsewhere [34–37].

Clearly, the issue of optimum antimicrobial chemotherapy of CAP remains to be resolved and will be dependent on the acquisition of data from large, well-controlled multicentre, randomised clinical trials [37–39]. One such trial, the CAP-START study, is currently underway in Holland [38, 40]. This is a “multicentre, cluster randomised crossover trial” involving seven Dutch hospitals and 2100 hospitalised nonintensive care unit (ICU) patients. It is designed to compare the therapeutic efficacy of (i) *β*-lactam monotherapy; (ii) *β*-lactam/macrolide combination; and (iii) fluoroquinolone monotherapy. The primary outcome is all-cause mortality 90 days after hospital admission [38, 40].

### 1.6. Antimicrobial Resistance

There is a considerable body of literature devoted to the issue of antimicrobial resistance among the common pathogens causing CAP, especially with regard to the pneumococcus [41–44]. Many studies describing the burden of disease caused by CAP confirm that there is emerging and increasing resistance among many of the CAP pathogens, a phenomenon that is occurring worldwide and which involves all classes of antimicrobial agents, to a greater or lesser extent [2–5, 41]. Accordingly, it has been recommended that the choice of empiric antimicrobial therapy for patients with CAP must be based on prediction of the most likely infecting pathogens together with a full appreciation of the common antimicrobial susceptibility patterns of these pathogens in a given geographical area (unit, ward, or practice).

While it is clear that there is emerging resistance among the CAP pathogens, and particularly the pneumococcus [42], it remains unclear whether the presence of antimicrobial resistance alone is associated with unfavourable treatment outcomes [41]. There have been a number of studies and various reviews discussing the issue of antibiotic resistance in the management and outcome of pneumococcal CAP [41, 45–49]. While there have been some studies that have purported to document a higher mortality rate in patients infected with penicillin-resistant compared with penicillin-susceptible pneumococcal pneumonia [47], most other studies or reviews have not reached similar conclusions [41, 45, 46, 48, 49].

In a large study of 844 hospitalised cases with bacteremic pneumococcal CAP, the authors failed to document penicillin resistance as a risk factor for mortality [45]. Furthermore, these authors noted that discordant therapy (receipt for the first 2 days after the positive blood culture of a single antibiotic that was inactive against the pneumococcus isolated) with penicillin, cefotaxime, or ceftriaxone did not result in a higher mortality rate. Others has confirmed that resistance to penicillins and third-generation cephalosporins have not been associated with significant increased mortality [48]. One critical review of the literature documented only a single microbiological failure of a parenteral penicillin-class antibiotic in the treatment of a patient with pneumococcal pneumonia [49]. Some have suggested that the patients who are at risk for antibiotic-resistant pathogens (due to conditions such as advanced age, or underlying comorbid conditions) are those that may already have host risk factors for a higher mortality [42]. It is, however, important to recognise that changes in the breakpoint definitions for the penicillins and third-generation cephalosporins have occurred since that time [50] such that many of those pathogens previously described as penicillin-resistant would now be considered susceptible. Nevertheless, the previous recommendations that therapy in these patients should continue with high-dose penicillins and broad-spectrum cephalosporins is still recommended in current guidelines [46, 49].

The situation with macrolides and fluoroquinolones is less clear-cut [41, 46]. Macrolide resistance may be of low level, associated with an efflux mechanism (*mef* gene), or of high level, associated with ribosomal target site mutations (*erm* gene), and while the latter has clearly been associated with treatment failure, there have also been some cases of failure with the former, although relatively small in number [41, 46]. For this reason, it has been recommended that awareness of pneumococcal macrolide resistance levels and patterns in a given region, as well as the risk factors in individual patients for macrolide resistance, clearly determine the utility of macrolide monotherapy in the management of pneumococcal CAP. With the respiratory fluoroquinolones, it is clear that laboratory documented resistance is likely to be associated with clinical failure, but what is less well known is that organisms documented in the laboratory as being susceptible, sometimes harbour one-step mutations in their quinolone resistance-determining regions that may undergo further mutations on therapy that may render them resistant [41].

Clearly new options for the treatment of antibiotic resistant pneumococcal infections are desirable, and to this end several newer agents have recently been introduced which have enhanced activity against resistant pneumococcal infections. This topic has been reviewed elsewhere and includes a potential role for ceftaroline, linezolid, telavancin, and tigecycline [51].

### 1.7. Severity of Illness

The severity of the infection dictates a number of important issues in the management of patients with CAP. Severity of illness determines the site of care (in- or outpatient), the extent of the microbiological workup, and the choice of initial empiric antimicrobial therapy [6]. Increased severity of infection is associated with greater healthcare needs and costs. While to a large extent assessment of severity of infection is still based primarily on sound clinical judgment, researchers have been attempting to develop mechanisms by which severity may be objectively assessed, such as the use of clinical scoring systems, various biomarkers, or by measuring microbial load.

#### 1.7.1. Severity of Illness Scoring Systems

A number of severity of illness scoring indices have been developed to assist in the evaluation of severity of pneumonia, of which the most commonly used are the Pneumonia Severity Index (PSI) and the CURB-65 [6, 7, 52, 53]. The PSI uses 20 variables which include patient age, gender, presence or absence of comorbid conditions, and/or vital sign abnormalities, as well as various laboratory and radiographic parameters [6, 54]. The CURB-65 uses only 5 variables, namely, presence or absence of confusion, urea >7 mmol/L, respiratory rate ≥30 breaths/minute, low blood pressure (systolic <90 mmHg or diastolic ≤60 mmHg), and age ≥65 years [6, 54]. With both scoring systems, cases can be stratified into low-, moderate-, or high-risk groups. The PSI was developed primarily to assist in identifying those patients who could safely be managed at home, whereas the CURB-65 was developed to document patients that were more severely ill, including those who needed to be flagged for ICU admission [55]. One of the advantages of the CURB-65 scoring system is that it is easy to use, but a limitation is that it may underestimate pneumonia severity in younger patients with comorbidities [55]. The PSI has been well validated and performs well in the assessment of low mortality risk patients, but it is complex to calculate and requires the evaluation of a number of laboratory parameters that potentially limits its use outside a hospital [55].

A number of additional scoring systems have also been developed, including those designed for the assessment of more severely ill cases, particularly those cases flagged for ICU admission, including the IDSA/ATS criteria, SMART-COP, PIRO-CAP, and SCAP, which appear to have better discriminatory values than PSI or CURB-65 in this situation [7, 54–56]. Each of the scoring systems that have been developed has various strengths and potential weaknesses so that no system is ideal, being unable to identify all patients at risk, or to replace clinical judgment; nonetheless, they are useful adjuncts for assessing cases [6, 53, 55]. Also of concern is that the scoring systems have been developed for assessment of patients on admission only and there is clearly a need for evaluating patients during the course of their hospitalisation, particularly in the setting of clinical deterioration [53].

#### 1.7.2. Biomarkers

More recently, a number of biomarkers has been tested to determine their ability to stratify risk in patients with CAP, sometimes as an adjunct to clinical scoring systems [7, 55]. Among these are inflammatory markers, such as the white blood cell count, acute phase reactants, such as C-reactive protein (CRP), cytokines, such as interleukin-1 and tumour necrosis factor-*α*, stress hormones, and various other molecules [6, 53, 55]. Of these, C-reactive protein (CRP) and procalcitonin (PCT) have been particularly well studied and have been reported in many studies to be useful tools, sometimes with an accuracy similar to that of CURB-65 or one of the other scoring systems, increasing the accuracy of severity assessment when used in combination with these scoring systems [7, 55, 57]. CRP, for example, is universally available and in some studies has been shown to have value in site-of-care decisions in predicting 28-day mortality, and in the prediction of treatment failure [7, 55]. PCT has been shown in some studies to be more useful than CRP in predicting severity and outcome in patients with CAP but is not widely available in many healthcare systems, possibly because of its much higher cost [7, 55]. It has been said that PCT should be considered to be a prognostic indicator rather than a diagnostic factor, but there is evidence that it may safely help reduce the unnecessary use of antibiotics, reducing bacterial resistance and curbing healthcare costs without increasing mortality [57–60]. Other biomarkers include proatrial natriuretic peptide (proANP), B-type natriuretic peptide (BNP), provasopressin (proVP), adrenomedullin (ADM), proadrenomedullin (proADM), arginine vasopresine (AVP), cortisol, D-dimers, copeptin, and soluble triggering receptor expressed on myeloid cells-1 (TREM-1) [7, 53, 55].

#### 1.7.3. Bacterial Load

A number of recent studies has documented that the pneumococcal bacterial DNA load correlates well with the severity of infection and has prognostic value, thus confirming a concept that has long been proposed, and which has become more accurate with the acquisition of more reliable assays [41, 47]. It has also been suggested that repeated measurements of the bacterial DNA load can accurately monitor treatment progress; however, more studies are needed to confirm these findings [41].

### 1.8. Clinical Outcome and Mortality

#### 1.8.1. Clinical Stability/Failure

The evaluation of the clinical outcome of a patient with pneumonia is an important aspect of medical care and has been reviewed elsewhere [61]. Achievement of clinical stability dictates important management issues such as change from intravenous to oral therapy, the timing of hospital discharge, and assessment of likely patient outcome. A number of risk factors for clinical failure have been identified, but most do not recognize pneumococcal aetiology as an important issue [61]. For example, in one study even the presence of pneumococcal bacteraemia did not increase the risk of poor outcome [62].

#### 1.8.2. Mortality

Respiratory tract infections, of which CAP plays a large role, are a major cause of death worldwide [8]. While many of the studies of CAP mortality have concentrated on in-hospital or 30 day mortality, others have evaluated long-term mortality (e.g., over a one-year period). One such study in older patients noted a hospital mortality of 11% versus 5.5% in controls (case controls matched for age, gender, race), while the one-year mortality was 40.9% versus 29.1%, respectively [52, 63]. Prior to that and subsequently, there has been a myriad of additional studies documenting high long-term mortality in patients with CAP [64]. One study documenting high long-term morbidity and mortality in CAP patients noted that it occurred particularly in those cases with high initial PSI scores [65]. Although many predisposing mechanisms have been documented, the presence of cardiovascular disease [66] and other comorbid conditions, including HIV infection, the subsequent documentation of primary or secondary neoplasms, and alterations in immune function predominate [64]. Cardiovascular events are among the most studied, with a number of reports documenting an increased risk of cardiovascular events during and after serious infections, such as CAP [64, 67–69].

The remaining sections of this review are focused on (i) the major virulence determinants of the pneumococcus, the most common cause of CAP, and their involvement in triggering harmful inflammatory responses [70–113] and (ii) control of these using pharmacological, anti-inflammatory strategies.

## 2. Virulence Determinants of *S. pneumoniae*

*S. pneumoniae* colonises the human nasopharynx during the first few months of life. The human airway employs numerous mechanisms to protect the airways from colonisation and invasive pneumococcal infection. In the case of innate immune defences, these include the cough reflex, the mucociliary escalator, and a range of pattern recognition receptors (PRRs) [100]. The PRRs include Toll-like receptors (TLRs), nucleotide-binding oligomerisation domain receptors (NOD-like receptors, NLRs), RIG-1-like receptors (RLRs), and the manifold cytosolic DNA sensors (reviewed in [114]). PRRs recognize pathogen-associated molecular patterns (PAMPs). In addition, infected cells, or cells which are stressed, release host-derived molecules known as damage-associated molecular patterns (DAMPs) or alarmins. During an infection, DAMPs and PAMPs have been shown to synergize leading to synthesis and secretion of proinflammatory cytokines and chemokines, including IL-1*β* and IL-8, as well as stimulating cell differentiation and cell death [115].

Although these mechanisms protect the airways from *S. pneumoniae*, antibody-mediated mechanisms, and cell-mediated immunity are critical in clearing the lower airways of this pathogen. Cell-mediated resolution of nasopharyngeal colonisation involves both Th1 and Th17 responses [116, 117] triggered by a variety of surface virulence determinants such as the bacterial capsule, pili, and other adhesins, as well as the toxin, pneumolysin.

The major virulence determinants and mechanisms of subversion of host defences by *S. pneumoniae* are summarized in Table 1 [70–113]. The most significant of these virulence factors are described in more detail below.

### 2.1. Capsular Polysaccharide

The anti-phagocytic polysaccharide capsule is considered to be the main determinant of pneumococcal virulence and is essential for colonisation of the nasopharynx [71, 72, 84]. Ninety-two different capsular serotypes have been identified [100]. Capsular morphology alternates between two distinct phases known as the transparent and opaque phenotypes [118]. Relative to the opaque variant, the transparent phenotype has decreased capsular thickness and expresses less pneumococcal surface protein A (PspA) in the setting of a higher level of expression of the major adhesin, choline-binding protein A (CbpA), and the autolysin, LytA, favouring adhesion and colonisation [119]. A further decrease in capsular thickness precedes the transition from colonisation to invasion of the epithelium [120, 121]. The opaque form is the main variant found in the circulation. This highly phagocytosis-resistant variant has increased capsular thickness and expression of PspA, in the setting of decreased expression of CbpA, favouring extrapulmonary dissemination [119].

Cell-wall fragments and capsular polysaccharides of *S. pneumoniae* are recognised and bound by antibodies which in turn bind complement [84], with C1q binding correlating closely with the deposition of C3b and C3bi on the bacterial surface [122]. Capsular serotypes differ with respect to invasiveness, due mainly to differences in complement deposition on the capsular polysaccharide, as well as binding of complement factor H [123].

*S. pneumoniae* has been found to grow in chains of variable length and, in addition to capsular polysaccharide, longer chains appear to favour adherence and colonisation [124].

### 2.2. Pneumococcal Pilus

Pili also promote pneumococcal virulence. These are only expressed by certain strains and enable the bacteria to survive in the lung and to bind to epithelial cells [74]. Pileated strains also induce a greater TNF-dependent inflammatory response, increasing the potential to produce lung injury and invade host tissue [73].

### 2.3. Biofilm

Although the ability of *S. pneumoniae* to grow and persist as biofilms does not appear to reflect virulence potential [125], it remains advantageous, as biofilm-encased bacteria show a reduced susceptibility to antimicrobial agents and resistance to immune recognition. Domenesch and colleagues have shown that there is reduced phagocytosis of pneumococcal biofilms due to impaired deposition of C3b. Biofilm formation by *S. pneumoniae* is also effective in preventing not only activation of the classical complement pathway due to reduced binding of C-reactive protein and the complement component C1q, but also by suppressing the pneumococcal surface protein C-(PspC-) dependent activation of the alternative complement pathway [78].

Recent studies suggest that biofilms do not contribute to the development of invasive pneumococcal disease, but, rather appear to confer a quiescent mode of growth during colonisation [101]. The role of the capsular polysaccharide in biofilm formation has not yet been determined conclusively, although the absence of the capsular polysaccharide was reported in one study to favour biofilm formation [126]; however, another study showed that decreased capsular polysaccharide formation was associated with decreased biofilm production [78]. A recent study has shown that augmentation of pneumococcal biofilm formation due to cigarette smoking is likely to favour microbial colonisation and persistence [127].

### 2.4. Hydrogen Peroxide (H_2_O_2_)

The pneumococcus is a major producer of H_2_O_2_ as a consequence of the activity of pyruvate oxidase, which is surprising, given that this catalase-negative pathogen is ill equipped to detoxify this oxidant [128]. The pneumococcus not only utilises H_2_O_2_ as a virulence factor, causing significant damage to ciliated respiratory epithelium and impaired protective activity of the mucociliary escalator [79], but also to eliminate microbial competitors in the nasopharynx [81]. In addition, pyruvate oxidase has been reported to act as a sensor of the oxygenation status of the microbial environment, regulating both nutritional capability and thickness of the anti-phagocytic capsule [129].

### 2.5. Surface Proteins

The adherence and colonisation of host tissues by the pneumococcus is mediated by surface adhesins and enzymes [83, 84] which are also important virulence factors of *S. pneumoniae*. The most significant of these are described below.

#### 2.5.1. Autolysins

Autolysins are cell-wall degrading proteases. These enzymes break down the peptidoglycan backbone enabling cell growth and cell division. However, excessive activity of autolysins results in the degradation of the cell-wall leading to cell lysis [86, 87]. *N*-acetylmuramic acid L-alanine amidase, also known as LytA amidase, is the major autolysin of *S. pneumoniae* [130]. The lysis of a portion of the bacterial cell-wall by LytA may increase the virulence of *S. pneumoniae* by promoting the release of potentially lethal toxins such as pneumolysin [84, 86].

Three other cell-wall hydrolytic enzymes have been identified *viz*. LytB, LytC, and choline binding protein E (CbpE) [84], all of which have been associated with nasopharyngeal colonisation [88].

#### 2.5.2. Choline Binding Protein A

Choline binding proteins (Cbp) have a C-terminal binding module followed by a flexible proline-rich segment and a functional N-terminal module [83]. CbpA binds to terminal choline residues of teichoic or lipoteichoic acids present on the surface of *S. pneumoniae*, anchoring the pathogens to human cell glycoconjugates, favouring the transition from colonisation to invasion [83].

#### 2.5.3. Pneumococcal Surface Protein A

Pneumococcal surface protein A (PspA) is also an important virulence factor of *S. pneumoniae*, inhibiting deposition of C3b on the bacterial surface, thereby interfering with complement activation and complement-dependent phagocytosis [91]. PspA also binds to and interferes with lactoferrin, increasing the availability of free iron required for bacterial growth [92].

#### 2.5.4. Pneumococcal Adherence and Virulence Factors A and B

The adhesins, PavA and B of *S. pneumoniae*, promote the invasion of host cells and dissemination of the pneumococcus. PavA has been shown to bind to the extracellular matrix component, fibronectin, while PavB binds both fibronectin and plasminogen. These adhesive effects are mediated by repetitive sequences designated streptococcal surface repeats (SSURE) [95, 96]. It has been suggested that PavA may affect pneumococcal colonisation by modulating the expression or function of virulence factors of *S. pneumoniae* [95].

#### 2.5.5. Neuraminidase

Three forms of neuraminidase have been identified in pneumococci, these being designated as NanA [105, 131], NanB [104, 131], and NanC [131]. NeuraminidaseA cleaves terminal sialic acid from cell surface glycans such as mucin, glycolipids, and glycoproteins [131], with resultant exposure of binding sites on the host cell surface contributing to pneumococcal adhesion and colonisation [83, 104, 105, 132]. NanA also plays an important role in biofilm formation [92, 95]. NanB is involved in the metabolic utilisation of sialic acid as a carbon and energy source by the pneumococcus, while NanC has a regulatory role [131, 133].

#### 2.5.6. Hyaluronidase

Hyaluronidase is secreted by pneumococci and breaks down the hyaluronic acid component of host connective tissue and extracellular matrix [84]. Increased epithelial permeability caused by the action of hyaluronidase favours the spread and colonisation of *S. pneumoniae* [84], especially when acting in concert with pneumolysin [134].

#### 2.5.7. Pneumolysin

The pneumococcal protein toxin, pneumolysin, is a member of the family of thiol-activated cytolysins and a critical virulence factor of the pathogen [85]. The toxin binds to cholesterol in the cytoplasmic membrane of eukaryotic cells, followed by insertion into the membrane, leading to the formation of large pores and cytolysis [83, 84]. In the early stages of infection, pneumolysin promotes nasopharyngeal colonisation via its inhibitory effects on ciliated respiratory epithelium [83]. In addition, the toxin has been shown to disrupt tight junctions thereby disrupting the integrity of the epithelial monolayer favouring invasiveness of the pathogen [82].

At high, cytotoxic concentrations, pneumolysin may also inhibit the protective functions of cells of both the innate and adaptive immune systems, as well as maturation of dendritic cells [135]. However, at lower noncytolytic concentrations, the toxin possesses proinflammatory activity as a consequence of sublytic pore formation and influx of Ca^2+^ into immune and inflammatory cells. This, in turn, causes hyperactivation of phagocytes, induction of proinflammatory cytokine production, and activation of the inflammasome, all of which are potentiated by the complement-activating properties of the toxin (as described below).

## 3. Harmful Effects of Excessive Activation of Antipneumococcal Host Defences

During pneumococcal CAP, a high bacillary load, aggravated by the implementation of antimicrobial chemotherapy with bactericidal agents which promote disintegration of the pathogen, results in excessive release of pro-inflammatory bacterial cell-wall products, toxins, and DNA. The consequence of these events is hyperactivation of host defence mechanisms, posing the potential threat of inflammation-mediated pulmonary damage and extrapulmonary spread of the pneumococcus. The major contributors to overexuberant inflammatory responses include (i) the pro-inflammatory, pore-forming interactions of pneumolysin with neutrophils, macrophages, and epithelial cells, potentiated by complement-activating properties of the toxin; (ii) the interactions of pneumolysin, lipoteichoic acid, proteoglycan, and DNA with PRRs, especially on cells of the innate immune system and epithelial cells; (iii) inappropriate induction of neutrophil extracellular trap (NET) formation by pneumococcal *α*-enolase and possibly pneumolysin and H_2_O_2_; and (iv) possible inappropriate oxidative activation of redox signalling mechanisms in immune and inflammatory cells by pneumococcal H_2_O_2_.

### 3.1. Pneumolysin-Mediated Mechanisms

In addition to directly causing acute lung injury as a consequence of its cytotoxic effects on airway epithelium and endothelium [136], pneumolysin, via its complement-activating activities and sub-lytic, pore-forming interactions with neutrophils and macrophages, also potentiates the release of reactive oxygen species (ROS), granule proteases, leukotriene B4, and prostaglandin E2 by promoting the movement of extracellular Ca^2+^ into the cells [137, 138]. As a result of Ca^2+^ influx, several intracellular signalling cascades are activated. These involve p38 and mitogen-activated protein kinases, transforming growth factor-*β*-activated kinase 1-mitogen-protein kinase 3/6-p38 *α*/*β*, Ca^2+^-calcineurin, nuclear factor kappa B (NF*κ*B), and activator protein 1 (AP-1) [139–142]. The consequence is increased production of IL-8 and TNF, both of which promote neutrophil influx into the airways [140–143]. In addition, pneumolysin also activates the NLRP3 inflammasome in dendritic cells, and presumably other immune and inflammatory cell types, thereby potentiating caspase-1-mediated conversion of pro-IL-1*β* to the mature cytokine [144].

The aforementioned direct cytolytic actions of pneumolysin, acting in concert with the indirect proinflammatory activities of the toxin, promote the epithelial/endothelial damage which favours dissemination of the pneumococcus.

### 3.2. Pattern Recognition Receptors

The interactions of the pneumococcus with the various PRRs expressed by cells of the innate immune system, as well as epithelial cells, have been the subject of a recent review [114]. Pneumococcal cell wall components, pneumolysin, and DNA have all been reported to interact with, and activate, several different types of PRRs. In the case of the Toll-like receptors (TLRs), lipoteichoic acid, and, possibly proteoglycan, are TLR-2 ligands [114], pneumolysin has been reported to interact with and activate TLR-4 [145], while pneumococcal CpG-motif-containing DNA is detected by TLR-9 [114]. In each case, triggering of TLRs is accompanied by activation of NF*κ*B and synthesis of proinflammatory chemokines/cytokines, especially IL-8 and TNF [114].

In addition, pneumococcal proteoglycans are recognised by and activate the nucleotide oligomerisation domain-like receptor, Nod2 [114], while microbial DNA is detected by the abundant cytosolic sensors of pathogen-derived nucleic acid [146, 147]. The consequence of these events is activation of NF*κ*B, as well as the interferon regulatory transcription factors 3 and 7 (IRF 3/7) [114].

### 3.3. Induction of Neutrophil Extracellular Traps (NETs)

NET formation is a postactivation strategy used mainly by dead and dying neutrophils and several other cell types including monocytes/macrophages and eosinophils, to isolate and kill microbial pathogens by trapping them in an extracellular matrix of citrullinated histones impregnated with antimicrobial granule proteins [148]. In the case of the pneumococcus, pathogen-derived *α*-enolase has been reported to induce NET formation following exposure of neutrophils to the pathogen [149]. Although unproven, it seems probable that pneumolysin and pathogen-derived H_2_O_2_ are also potential inducers of NET formation. However, as opposed to being an effective antipneumococcal host defence mechanism, the pneumococcus appears to be particularly adept at escaping from NETs [150], apparently as a consequence of production of the endonuclease, End A, which mediates degradation of NETs [107]. In addition to subverting NETs, the pneumococcus may also exploit poorly regulated NET formation as a strategy to promote invasion and dissemination due to epithelial and endothelial cytotoxicity mediated by the histone components of NETs [151].

### 3.4. Hydrogen Peroxide

While its role in microbial virulence is well established, the involvement of pneumococcus-derived H_2_O_2_ in activating harmful inflammatory responses during CAP and other infections remains to be established. This seems likely, however, as cell-permeable H_2_O_2_ is a potent activator of redox intracellular signalling mechanisms in many cell types, including those of the innate and adaptive immune systems [152, 153].

These various mechanisms of hyperactivation of inflammatory responses operative during pneumococcal CAP are summarised in Table 2.

## 4. Adjunctive Anti-Inflammatory Therapeutic Strategies in Severe CAP

The primary objective of adjunctive pharmacological strategies in severe CAP, especially severe pneumococcal disease, is to suppress overexuberant, harmful, pathogen-activated inflammatory responses, thereby attenuating inflammation-mediated pulmonary damage and dysfunction. In this setting, the three categories of anti-inflammatory agents which have attracted the greatest interest are macrolides, corticosteroids, and, more recently, statins [31, 32, 34–39]. Other categories of anti-inflammatory agent which remain largely untested, include the various types of 3′-5′-cyclic adenosine monophosphate- (cAMP-) elevating agents, as well as non-steroidal anti-inflammatory agents (NSAIDs) [34].

### 4.1. Macrolides

Despite the absence of irrefutable proof of efficacy, the inclusion of macrolides in the current guideline recommendations for the therapy of CAP can be justified on several grounds. Notwithstanding their primary antimicrobial activities, which complement those of *β*-lactams in the treatment of CAP, macrolides possess well-recognised anti-inflammatory properties. These are both pathogen- and host-directed and have recently been reviewed in detail elsewhere [34, 35]. Briefly, the pathogen-targeted anti-inflammatory activity of macrolides is achieved via the inhibitory effects of these agents on bacterial protein synthesis, thereby attenuating the production of pro-inflammatory toxins, such as pneumolysin in the case of the pneumococcus [35, 154]. In addition, abrupt bacteriolysis and accompanying excessive inflammation due to release of cell-wall components and endotoxins, as may occur with bactericidal antibiotics, are also countered by the predominantly bacteriostatic activity of macrolides [35].

The primary target of the secondary anti-inflammatory properties of macrolides, unrelated to antimicrobial activity, is neutrophil recruitment. Macrolide-mediated inhibition of neutrophil mobilisation is achieved predominantly via inhibition of production of the neutrophil-mobilising cytokines/chemokines IL-8, IL-17, and TNF, not only by cells of the innate immune system, but also by various types of structural cells [35, 155]. Although not fully understood, these inhibitory effects of macrolides on the synthesis of pro-inflammatory cytokines/chemokines appear to be achieved at the level of gene transcription. This results from antagonism of transcription factors such as nuclear factor kappa B (NF*κ*B), possibly via (i) interference with redox signalling mechanisms; and (ii) enhancement of histone deacetylase activity [35, 156–158].

The beneficial therapeutic activities of these various pathogen- and host-directed anti-inflammatory activities of macrolides are evident in several chronic inflammatory diseases of the airways, particularly bronchiolitis obliterans, diffuse panbronchiolitis, and cystic fibrosis, and possibly chronic obstructive pulmonary disease (reviewed in [35]). Although difficult to prove conclusively, several lines of evidence, both clinical and experimental, also support the involvement of the anti-inflammatory activities of macrolides in controlling severe sepsis and/or inflammation-mediated lung injury in acute bacterial infection. This contention is supported by at least two noteworthy studies. The first of these reported that the use of macrolides was associated with decreased mortality in patients with pneumonia and severe sepsis, even in patients infected with macrolide-resistant pathogens, such as gram-negative organisms [33]. The second study, which was undertaken in patients with predominantly macrolide-resistant gram-negative sepsis and ventilator-associated pneumonia (VAP), reported that intravenous administration of clarithromycin (1 gram/daily) for 3 days resulted in accelerated resolution of VAP and earlier discontinuation of mechanical ventilation, as well as delaying, but not preventing, mortality [159]. More recently, administration of a macrolide within 24 hours of trial entry, but not *β*-lactam or fluoroquinolone antibiotics, to patients with acute lung injury secondary to pneumonia in most cases, was associated with significant decreases in both 180 day mortality and time to successful discontinuation of mechanical ventilation [160].

The beneficial anti-inflammatory activities of macrolides and macrolide-like antimicrobial agents have also been demonstrated in various animal models of experimental chemotherapy of acute bacterial infection (reviewed in [35]). In one such study reported by Karlström et al., using a murine model of pneumococcal pneumonia secondary to influenza virus infection, treatment of animals with either azithromycin or clindamycin alone or in combination with ampicillin resulted in significantly improved survival compared to animals treated with ampicillin only [161]. These beneficial effects of azithromycin/clindamycin were associated with decreased concentrations of airway pro-inflammatory cytokines and influx of inflammatory cells in the setting of less severe histopathological changes [161].

Because macrolides combine pathogen- and host-directed anti-inflammatory activities, therapy with these agents appears to be an ideal adjunctive strategy in severe CAP, possibly in a subset of patients at risk for development of ALI. Nonetheless, widespread acceptance of an adjunctive role for macrolides in this clinical setting is dependent on the acquisition of compelling data from prospective, randomised, controlled clinical trials [36–39, 162, 163].

### 4.2. Corticosteroids

Corticosteroids (CS) are broad-spectrum anti-inflammatory agents, but unlike macrolides, they are less effective in targeting neutrophils [164]. Although an adjunctive role for systemic CS in the clinical management of adults with penicillin-susceptible pneumococcal meningitis is well recognised [165], their role in the adjunctive therapy of severe CAP remains unproven. Several relatively small prospective/retrospective trials conducted between 2005 and 2007 in hospitalised patients with severe CAP, receiving systemic CS at doses varying from 40 to 200 mg/daily for periods of 3 days and longer, reported beneficial effects of these agents on, amongst others, duration of hospital stay, and mortality [166–168]. However, this adjunctive promise of systemic CS was not confirmed in several more recent studies. In a large randomised, double-blinded, placebo-controlled trial, Snijders et al. failed to detect beneficial effects of systemic administration of prednisone (40 mg/daily for 7 days) on outcome in patients with CAP, with the frequency of late failure (>72 hours after hospital admission) being significantly (*P* < 0.04) more common in CS-treated patients [169]. In agreement with these findings, Polverino et al., in a retrospective study covering an approximately 10.5 year period involving 3257 patients who received a mean systemic CS daily dose of 45 mg for varying periods of time, reported that administration of CS did not influence either clinical stability or mortality [170]. Somewhat worryingly, however, administration of CS significantly prolonged hospital stay (9 versus 6 days, *P* < 0.01) [170].

In contrast, Meijvis et al. reported that systemic administration of dexamethasone, at the comparatively low dose of 5 mg/daily for 4 days from the time of admission, to nonimmunocompromised patients who did not require ICU admission, was associated with a significant decrease in length of hospital stay (7.5 versus 6.5 days *P* < 0.048) [171]. These observations suggest that the dose of the systemic CS and the immune status of the patient are potential determinants of the success of adjunctive therapy with CS in patients with CAP. In addition, the type of pathogen may also be a determinant of successful outcome of CS therapy, with infections caused by atypical pathogens, as opposed to those caused by *S. pneumoniae,* being more responsive to the beneficial actions of CS [172].

However, as concluded in a recent meta-analysis, conclusive proof of the role, if any, of systemic CS in the adjunctive therapy of severe CAP is dependent on the acquisition of compelling data from adequately powered randomised trials [173]. Three such studies are ongoing, one in the USA (Extended Steroids in CAPe-ESCAPe, projected completion date January 2017) [174] and two in Europe [38].

### 4.3. Statins

Statins is the collective term for a group of pharmacological inhibitors of the enzyme, 3-hydroxy-3-methylglutaryl coenzyme A reductase, used to control hypercholesterolemia in the prevention of cardiovascular diseases and stroke. In addition to their cholesterol-lowering properties, statins have also been reported to possess significant anti-inflammatory activities which have been attributed to two major mechanisms. Firstly, interference with the prenylation of the small G-proteins Rac, Ras, and Rho, thereby attenuating G-protein-coupled receptor cellular signalling and activation in a variety of cell types, including immune and inflammatory cells [175–177]. The consequence is decreased activation of NF*κ*B and resultant interference with the transcription of genes encoding various proinflammatory proteins such as inducible nitric oxide synthase, cyclooxygenase-2 and matrix metalloproteinase-9 [176, 178]. Secondly, via induction of heme oxygenase-1 expression, also resulting in attenuation of activation of NF*κ*B, as well as decreased production of ROS in the setting of increased production of anti-inflammatory IL-10 [178, 179].

Interestingly, a number of predominantly retrospective studies conducted between 2005 and 2011 have reported that statin use in the prevention of cardiovascular diseases is associated with improved outcome of patients with CAP, possibly as a consequence of the anti-inflammatory activities of these agents. In the majority of these studies, which have recently been reviewed by Corrales-Medina and Musher [36], statin use was associated with an approximately 50% reduction in mortality. In addition to these, Doshi et al. in a retrospective study of patients (*n* = 347) with pneumococcal pneumonia, who presented at a single medical center between 2000 and 2010, reported that statin use, as opposed to administration of macrolides, was associated with decreased mortality 4, 14, 20, and 30 days after admission [39]. These findings suggest a mechanism in addition to those mentioned above by which statins may protect against invasive pneumococcal diseases. By decreasing plasma membrane concentrations of cholesterol in epithelial, endothelial, and immune/inflammatory cells, statins may restrict the binding of pneumolysin, thereby attenuating both the cytotoxic and pro-inflammatory activities of the toxin.

Although of considerable potential importance, the aforementioned studies have several significant limitations: (i) they do not address the adjunctive potential of statins administered at the time of presentation with CAP; (ii) concomitant use of statins may obscure the therapeutic potential of macrolides and CS; and (iii) statin use may be associated with a “healthy user” effect, distinguishing a subgroup of patients who are likely to have a better outcome [157]. Once again, large randomised, prospective, controlled clinical trials are necessary to evaluate the use of statins as a potential adjunctive therapy in CAP [36, 39].

### 4.4. Other Potential Adjunctive Therapies

Other largely untested adjunctive therapies in the clinical setting of CAP include (i) pharmacological agents which increase intracellular concentrations of the cyclic nucleotide, cAMP, in immune and inflammatory cells and (ii) NSAIDs. Cyclic AMP has been described as being “the master regulator of innate immune cell function” [180]; consequently pharmacological agents which increase the intracellular concentrations of this cyclic nucleotide possess broad-spectrum anti-inflammatory activities. Agents falling into this category include cAMP-specific and nonspecific phosphodiesterase inhibitors, as well as agonists of *β*_2_-adrenoreceptors, adenosine A2_A_ receptors, and the E-type prostaglandin receptors, EP_2_ and EP_4_. Although the potential of these agents is untested in CAP, it is noteworthy that the *β*_2_-adrenoreceptor agonist, salbutamol, administered either intravenously or as an aerosol, has proved to be ineffective in the treatment of ALI [181, 182]. With respect to anti-inflammatory activity, salbutamol may not, however, be the most effective *β*_2_-agonist to use in this clinical setting [183].

Administration of the NSAID, naproxen, to healthy individuals has been reported to augment the bactericidal action of whole blood against a penicillin-resistant strain of *S. pneumoniae ex vivo *[184]. These potentiating effects of the NSAID were associated with increased phagocytic activity of blood phagocytes, as well as increased generation of antimicrobial ROS by these cells, due, presumably, to inhibition of production of immunosuppressive prostaglandin E_2_ and activation of adenylyl cyclase via EP_2_/EP_4_ receptors [184]. Somewhat paradoxically, however, these antipneumococcal actions of NSAIDs may predispose to inflammation-mediated tissue damage via interference with cAMP-mediated immunoregulatory activity.

The aforementioned adjunctive anti-inflammatory therapies in CAP are summarised in Table 3. These and other types of adjunctive therapies, largely unsuccessful, have also been the subject of several recent reviews and include (i) intravenous gammaglobulin (of unproven benefit); (ii) monoclonal antibodies targeted against the IL-1 or PAF receptors, as well as TNF; (iii) recombinant human activated protein C (drotrecogin alfa); and (iv) the recombinant tissue factor pathway inhibitor, tifacogin [34, 162].

## 5. Conclusion

It is quite evident from a review of the scientific literature that CAP, and in particular infection due to *Streptococcus pneumoniae, *carries a considerable burden of disease among the world's population. The pneumococcus is the most common microbial cause of CAP, not only in mild infections, but also among patients requiring hospitalisation and even among critically ill cases. The reason that pneumococcal infection and CAP remain so common throughout the world relates to the high prevalence of risk factors for this infection in the general population, which includes aging, lifestyle factors, and underlying comorbid illnesses and, at least in some parts of the world, concomitant HIV infection. Pneumococcal pneumonia causes considerable morbidity and mortality and treatment of these infections is potentially being compromised by the emergence of resistance in this microorganism to the commonly used antibiotics. The pneumococcus expresses a large number of virulence factors, which not only render the microorganism very effective in causing infection, but also contribute to disease pathogenesis via their cytotoxic and proinflammatory activities. Targeting these virulence factors additionally as part of overall therapy has the potential for improving the outcome of such infections.

## Figures and Tables

**Table 1:** Virulence determinants of *S. pneumoniae* and mechanisms of subversion of host defences.

Virulence factor	Function
Capsule	(i) Prevents entrapment in nasal mucus [70]
	(ii) Exhibits antiphagocytic activity [71]
	(iii) Facilitates adherence and colonisation of nasopharyngeal epithelial cells [72]
Pili	Enhances bacterial adhesion and ability to cause invasive disease [73, 74]
Pilus subunit (RrgA)	(i) Binds fibronectin, collagen I, and laminin [75]
	(ii) Prevents CR3-mediated phagocytosis [76]
	(iii) TLR2 agonist [77]
Biofilm	(i) Reduces susceptibility to antimicrobial agents [78]
	(ii) Prevents recognition and phagocytosis by the immune system [78]
H_2_O_2_	(i) Causes ciliary slowing and epithelial damage [79]
	(ii) Facilitates colonisation of nasopharyngeal epithelial cells [80]
	(iii) Bactericidal action against competing bacteria [81]
Pneumolysin	(i) Binds to cytoplasmic membrane cholesterol [82]
	(ii) Disrupts integrity of epithelial monolayer [83]
	(iii) Exhibits cytolytic activity [84]
	(iv) Modulates host inflammatory and immune responses [85]
Autolysin (LytA)	(i) Involved in autolysis resulting in the release of pneumolysin [86, 87]
	(ii) Facilitates colonisation of nasopharyngeal epithelial cells [88]
Choline binding protein A (CbpA)	(i) Promotes adhesion to human cell conjugates [83]
	(ii) Binds laminin [89]
Choline binding protein E (CbpE)	Mediates attachment to plasminogen [90]
Pneumococcal surface protein A (PspA)	(i) Inhibits complement-dependent phagocytosis [91]
	(ii) Binds lactoferrin [92]
Pneumococcal surface protein C (PspC)	Inhibits deposition of the terminal complement complex [93]
Pneumococcal adherence and virulence factor A (PavA)	Mediates attachment to plasminogen [94, 95]
Pneumococcal adherence and virulence factor B (PavB)	Mediates attachment to plasminogen and fibronectin [96]
Pneumococcal surface adhesin A (PsaA)	(i) Binds E-cadherin [97]
	(ii) Facilitates invasion of nasopharyngeal epithelial cells [98]
Plasmin and fibronectin binding protein A (PfbA)	Mediates attachment to plasminogen and fibronectin [99]
Pneumococcal serine-rich repeat protein (PsrP)	(i) Facilitates adherence to nasopharyngeal epithelial cells [100]
	(ii) Mediates biofilm production [101]
Putative histidine triad protein (PhpA)	Degrades C3 [102]
Neuraminidase (sialidase)	(i) Facilitates adherence and colonisation of nasopharyngeal epithelial cells [92, 103–105]
	(ii) Mediates biofilm production [103, 106]
Hyaluronidase	(i) Facilitates colonisation of nasopharyngeal epithelial cells [84]
	(ii) Aids the dissemination of the bacteria [84]
Endonuclease A (EndA)	Degrades neutrophil extracellular traps [107, 108]
Zinc metalloproteinase (ZmpB)	(i) Induces TNF production in the respiratory tract [109]
	(ii) Cleaves secretory IgA [110]
Streptococcus-specific glycosyl hydrolase (GHIP)	Facilitates invasion of nasopharyngeal epithelial cells [111]
ClpP protease	Induces apoptosis [112]
Ser/Thr kinase (StkP)	Regulator of cell division [113]

**Table 2:** Causes of overexuberant inflammatory responses during pneumococcal CAP.

Cause	Consequence
Excessive release of pneumolysin	Uncontrolled complement activation; hyperactivation of phagocytes and epithelial cells due to the noncytolytic, pore-forming actions of the toxin
Excessive release of bacterial cell-wall products (e.g., lipoteichoic acids and DNA), especially during chemotherapy with bactericidal agents	Sustained activation of various types of pathogen recognition receptors on/in cells of the innate immune system and epithelial cells, resulting in poorly regulated production of neutrophil-mobilising chemokines/cytokines
Poorly controlled formation of NETs with limited protective activity	Histone-mediated epithelial and endothelial toxicity, favouring extrapulmonary spread of the pneumococcus
Excessive release of cell-permeable, proinflammatory H_2_O_2 _by the pneumococcus	Uncontrolled activation of redox intracellular signalling mechanisms in cells of the innate and adaptive immune systems, as well as other cell types. The existence of this mechanism remains to be established

**Table 3:** Adjunctive anti-inflammatory therapies in CAP.

Type of adjunctive therapy	Current status
Macrolide antibiotics	Recommended in current guidelines primarily for antimicrobial activity. The clinical relevance of anti-inflammatory activity remains to be conclusively established
Corticosteroids	Remains controversial and is the subject of several ongoing randomised, prospective, controlled trials
Statins	Show promise, but therapeutic efficacy of initiation at the time of diagnosis of CAP remains to be established
cAMP-elevatory agents	Theoretically promising, although few safe and effective agents currently available; salbutamol found ineffective in the treatment of ALI
NSAIDs	Of questionable value

## References

[1] Feldman C, Brink AJ, Richards GA, Maartens G, Bateman ED (2007). Management of community-acquired pneumonia in adults. *South African Medical Journal*.

[2] File TM, Marrie TJ (2010). Burden of community-acquired pneumonia in North American adults. *Postgraduate Medicine*.

[3] Isturiz RE, Luna CM, Ramirez J (2010). Clinical and economic burden of pneumonia among adults in Latin America. *International Journal of Infectious Diseases*.

[4] Song J-H, Thamlikitkul V, Hsueh P-R (2011). Clinical and economic burden of community-acquired pneumonia amongst adults in the Asia-Pacific region. *International Journal of Antimicrobial Agents*.

[5] Welte T (2012). Risk factors and severity scores in hospitalized patients with community-acquired pneumonia: prediction of severity and mortality. *European Journal of Clinical Microbiology and Infectious Diseases*.

[6] Welte T, Torres A, Nathwani D (2012). Clinical and economic burden of community-acquired pneumonia among adults in Europe. *Thorax*.

[7] Brown JS (2012). Community-acquired pneumonia. *Clinical Medicine*.

[8] Fauci AS, Morens DM (2012). The perpetual challenge of infectious diseases. *The New England Journal of Medicine*.

[9] Blasi F, Mantero M, PierAchille S, Tarsia P (2012). Understanding the burden of pneumococcal disease in adults. *Clinical Microbiology and Infections*.

[10] Feldman C, Anderson R (2003). Cigarette smoking and mechanisms of susceptibility to infections of the respiratory tract and other organ systems. *Journal of Infection*.

[11] Sanz Herrero F, Blanquer Olivas J (2012). Microbiology and risk factors for community-acquired pneumonia. *Seminars in Respiratory and Critical Care Medicine*.

[12] Madeddu G, Laura Fiori M, Stella Mura M (2010). Bacterial community-acquired pneumonia in HIV-infected patients. *Current Opinion in Pulmonary Medicine*.

[13] Feldman C, Klugman KP, Yu VL (2007). Bacteraemic pneumococcal pneumonia: impact of HIV on clinical presentation and outcome. *Journal of Infection*.

[14] Feldman C, Anderson R (2013). HIV-associated bacterial pneumonia. *Clinics in Chest Medicine*.

[15] Martin-Loeches I, Solé-Violán J, Rodríguez de Castro F (2012). Variants at the promoter of the interleukin-6 gene are associated with severity and outcome of pneumococcal community-acquired pneumonia. *Intensive Care Medicine*.

[16] Dahmer MK, O’Cain P, Patwari PP (2011). The influence of genetic variation in surfactant protein B on severe lung injury in African American children. *Critical Care Medicine*.

[17] García-Laorden MI, Rodríguez de Castro F, Solé-Violán J (2011). Influence of genetic variability at the surfactant proteins A and D in community-acquired pneumonia: a prospective, observational, genetic study. *Critical Care*.

[18] Capelastegui A, España PP, Bilbao A (2012). Etiology of community-acquired pneumonia in a population-based study: link between etiology and patient characteristics, process-of-care, clinical evolution and outcomes. *BMC Infectious Diseases*.

[19] Cillóniz C, Ewig S, Menéndez R (2012). Bacterial co-infection with H1N1 infection in patients admitted with community acquired pneumonia. *Journal of Infection*.

[20] Martin-Loeches I, Sanchez-Corral A, Diaz E (2011). Community-acquired respiratory coinfection in critically ill patients with pandemic 2009 influenza A, (H1N1) virus. *Chest*.

[21] Rice TW, Rubinson L, Uyeki TM (2012). Critical illness from 2009 pandemic influenza A virus and bacterial coinfection in the United States. *Critical Care Medicine*.

[22] Alanee SRJ, McGee L, Jackson D (2007). Association of serotypes of *Streptococcus pneumoniae* with disease severity and outcome in adults: an international study. *Clinical Infectious Diseases*.

[23] Weinberger DM, Harboe ZB, Sanders EAM (2010). Association of serotype with risk of death due to pneumococcal pneumonia: a meta-analysis. *Clinical Infectious Diseases*.

[24] Garcia-Vidal C, Ardanuy C, Tubau F (2010). Pneumococcal pneumonia presenting with septic shock: host- and pathogen-related factors and outcomes. *Thorax*.

[25] Ahl J, Littorin N, Forsgren A, Odenholt I, Resman F, Riesbeck K (2013). High incidence of septic shock caused by **Streptococcus pneumoniae** serotype 3: a retrospective epidemiological study. *BMC Infectious Diseases*.

[26] Bewick T, Sheppard C, Greenwood S (2012). Serotype prevalence in adults hospitalised with pneumococcal non-invasive community-acquired pneumonia. *Thorax*.

[27] Benfield T, Skovgaard M, Schønheyder HC (2013). Serotype distribution in non-bacteremic pneumococcal pneumonia: association with disease severity and implications for pneumococcal conjugate vaccines. *PLoS ONE*.

[28] Johnstone J, Majumdar SR, Fox JD, Marrie TJ (2008). Viral infection in adults hospitalized with community-acquired pneumonia: prevalence, pathogens, and presentation. *Chest*.

[29] Huijskens EG, van Erkel AJ, Palmen FM, Buiting AG, Kluytmans JA, Rossen JW (2013). Viral and bacterial aetiology of community-acquired pneumonia in adults. *Influenza and Other Respiratory Viruses*.

[30] Mandell LA, Wunderink RG, Anzueto A (2007). Infectious Diseases Society of America/American Thoracic Society Consensus Guidelines on the management of community-acquired pneumonia in adults. *Clinical Infectious Diseases*.

[31] Martin-Loeches I, Lisboa T, Rodriguez A (2010). Combination antibiotic therapy with macrolides improves survival in intubated patients with community-acquired pneumonia. *Intensive Care Medicine*.

[32] Feldman C, Anderson R (2009). New insights into pneumococcal disease. *Respirology*.

[33] Restrepo MI, Mortensen EM, Waterer GW, Wunderink RG, Coalson JJ, Anzueto A (2009). Impact of macrolide therapy on mortality for patients with severe sepsis due to pneumonia. *European Respiratory Journal*.

[34] Feldman C, Anderson R (2011). Bacteraemic pneumococcal pneumonia: current therapeutic options. *Drugs*.

[35] Steel HC, Theron AJ, Cockeran R, Anderson R, Feldman C (2012). Pathogen- and host-directed anti-inflammatory activities of macrolide antibiotics. *Mediators of Inflammation*.

[36] Corrales-Medina VF, Musher DM (2011). Immunomodulatory agents in the treatment of community-acquired pneumonia: a systematic review. *Journal of Infection*.

[37] Asadi L, Sligl WI, Eurich DT (2012). Macrolide-based regimens and mortality in hospitalized patients with community-acquired pneumonia: a systematic review and meta-analysis. *Clinical Infectious Diseases*.

[38] Postma DF, van Werkhoven CH, Huijts SM, Bolkenbaas M, Oosterheert JJ, Bonten MJM (2012). New trends in the prevention and management of community-acquired pneumonia. *The Netherlands Journal of Medicine*.

[39] Doshi SM, Kulkarni PA, Liao JM, Rueda AM, Musher DM (2013). The impact of statin and macrolide use on early survival in patients with pneumococcal pneumonia. *The American Journal of Medical Sciences*.

[40] CAP-START study. http://clinicaltrials.gov/ct2/show/NCT01660204.

[41] Fuller JD, McGeer A, Low DE (2005). Drug-resistant pneumococcal pneumonia: clinical relevance and approach to management. *European Journal of Clinical Microbiology and Infectious Diseases*.

[42] Jones RN, Jacobs MR, Sader HS (2010). Evolving trends in *Streptococcus pneumoniae* resistance: implications for therapy of community-acquired bacterial pneumonia. *International Journal of Antimicrobial Agents*.

[43] Lynch JP, Zhanel GG (2010). *Streptococcus pneumoniae*: epidemiology and risk factors, evolution of antimicrobial resistance, and impact of vaccines. *Current Opinion in Pulmonary Medicine*.

[44] Feldman C, Anderson R (2012). Antibiotic resistance of pathogens causing community-acquired pneumonia. *Seminars in Respiratory and Critical Care Medicine*.

[45] Yu VL, Chiou CCC, Feldman C (2003). An international prospective study of pneumococcal bacteremia: correlation with *in vitro* resistance, antibiotics administered, and clinical outcome. *Clinical Infectious Diseases*.

[46] Feldman C (2004). Clinical relevance of antimicrobial resistance in the management of pneumococcal community-acquired pneumonia. *Journal of Laboratory and Clinical Medicine*.

[47] Ho PL, Que TL, Ng TK, Chiu SS, Yung RW, Tsang KW (2006). Clinical outcomes of bacteremic pneumococcal infections in an area with high resistance. *European Journal of Clinical Microbiology & Infectious Diseases*.

[48] Peterson LR (2006). Penicillins for treatment of pneumococcal pneumonia: does in vitro resistance really matter?. *Clinical Infectious Diseases*.

[49] Tleyjeh IM, Tlaygeh HM, Hejal R, Montori VM, Baddour LM (2006). The impact of penicillin resistance on short-term mortality in hospitalized adults with pneumococcal pneumonia: a systematic review and meta-analysis. *Clinical Infectious Diseases*.

[50] Clinical Laboratory Standards Institute Antimicrobial Susceptibility Testing M02-A10: Performance Standards for Antimicrobial Disk Susceptibility Tests.

[51] Rivera AM, Boucher HW (2011). Current concepts in antimicrobial therapy against select gram-positive organisms: methicillin-resistant *Staphylococcus aureus*, penicillin-resistant pneumococci, and vancomycin-resistant enterococci. *Mayo Clinic Proceedings*.

[52] Niederman MS (2007). Recent advances in community-acquired pneumonia: inpatient and outpatient. *Chest*.

[53] Kolditz M, Ewig S, Höffken G (2013). Management-based risk prediction in community-acquired pneumonia by scores and biomarkers. *European Respiratory Journal*.

[54] Feldman C (2007). Prognostic scoring systems: which one is best?. *Current Opinion in Infectious Diseases*.

[55] Pereira JM, Paiva JA, Rello J (2012). Assessing severity of patients with community-acquired pneumonia. *Seminars in Respiratory and Critical Care Medicine*.

[56] Marti C, Garin N, Grosgurin O (2012). Prediction of severe community-acquired pneumonia: a systematic review and meta-analysis. *Critical Care*.

[57] Lippi G, Meschi T, Cervellin G (2011). Inflammatory biomarkers for the diagnosis, monitoring and follow-up of community-acquired pneumonia: clinical evidence and perspectives. *European Journal of Internal Medicine*.

[58] Berg P, Lindhardt BØ (2012). The role of procalcitonin in adult patients with community-acquired pneumonia: a systemativ review. *Danish Medical Bulletin*.

[59] Schuetz P, Briel M, Christ-Crain M (2012). Procalcitonin to guide initiation and duration of antibiotic treatment in acute respiratory infections: an individual patient data meta-analysis. *Clinical Infectious Diseases*.

[60] Schuetz P, Müller B, Christ-Crain M (2012). Procalcitonin to initiate or discontinue antibiotics in acute respiratory tract infections. *Cochrane Database of Systematic Reviews*.

[61] Aliberti S, Blasi F (2012). Clinical stability versus clinical failure in patients with community-acquired pneumonia. *Seminars in Respiratory and Critical Care Medicine*.

[62] Bordón J, Peyrani P, Brock GN (2008). The presence of pneumococcal bacteremia does not influence clinical outcomes in patients with community-acquired pneumonia: results from the Community-Acquired Pneumonia Organization (CAPO) international cohort study. *Chest*.

[63] Kaplan V, Clermont G, Griffin MF (2003). Pneumonia: still the old man’s friend?. *Archives of Internal Medicine*.

[64] Mortensen EM, Metersky ML (2012). Long-term mortality after pneumonia. *Seminars in Respiratory and Critical Care Medicine*.

[65] Johnstone J, Eurich DT, Majumdar SR, Jin Y, Marrie TJ (2008). Long-term morbidity and mortality after hospitalization with community-acquired pneumonia: a population-based cohort study. *Medicine*.

[66] Corrales-Medina VF, Musher DM, Shachkina S, Chirinos JA (2013). Acute pneumonia and the cardiovascular system. *The Lancet*.

[67] Carr GE, Yeun TC, McConville JF (2012). Early cardiac arrest in patients hospitalized with pneumonia: a report from the American Heart Association’s Get With the Guidelines-Resuscitation Program. *Chest*.

[68] Corrales-Medina VF, Musher DM, Wells GA, Chirinos JA, Chen L, Fine MJ (2012). Cardiac complications in patients with community-acquired pneumonia incidence, timing, risk factors, and association with short-term mortality. *Circulation*.

[69] Soto-Gomez N, Anzueto A, Waterer GW, Restrepo MI, Mortensen EM (2013). Pneumonia: an arrhythmogenic disease?. *The American Journal of Medicine*.

[70] Kadioglu A, Weiser JN, Paton JC, Andrew PW (2008). The role of *Streptococcus pneumoniae* virulence factors in host respiratory colonization and disease. *Nature Reviews Microbiology*.

[71] Jonsson S, Musher DM, Chapman A (1985). Phagocytosis and killing of common bacterial pathogens of the lung by human alveolar macrophages. *Journal of Infectious Diseases*.

[72] Weinberger DM, Trzciński K, Lu YJ (2009). Pneumococcal capsular polysaccharide structure predicts serotype prevalence. *PLoS Pathogens*.

[73] Barocchi MA, Ries J, Zogaj X (2006). A pneumococcal pilus influences virulence and host inflammatory responses. *Proceedings of the National Academy of Sciences of the United States of America*.

[74] Bagnoli F, Moschioni M, Donati C (2008). A second pilus type in *Streptococcus pneumoniae* is prevalent in emerging serotypes and mediates adhesion to host cells. *Journal of Bacteriology*.

[75] Hilleringmann M, Giusti F, Baudner BC (2008). Pneumococcal pili are composed of protofilaments exposing adhesive clusters of Rrg A. *PLoS Pathogens*.

[76] Orrskog S, Rounioja S, Spadafina T (2012). Pilus adhesion RrgA interacts with complement receptor 3, thereby affecting macrophage function and systemic pneumococcal disease. *MBio*.

[77] Basset A, Zhang F, Benes C (2013). Toll-like receptor (TLR) 2 mediates inflammatory responses to oligomerized RrgA pneumococcal pilus type 1 protein. *Journal of Biological Chemistry*.

[78] Domenech M, Ramos-Sevillano E, Garcia E, Moscoso M, Yuste J (2013). Biofilm formation avoids complement immunity and phagocytosis of *Streptococcus pneumoniae*. *Infection and Immunity*.

[79] Feldman C, Anderson R, Cockeran R, Mitchell T, Cole P, Wilson R (2002). The effects of pneumolysin and hydrogen peroxide, alone and in combination, on human ciliated epithelium *in vitro*. *Respiratory Medicine*.

[80] Regev-Yochay G, Trzcinski K, Thompson CM, Lipsitch M, Malley R (2007). SpxB is a suicide gene of *Streptococcus pneumoniae* and confers a selective advantage in an *in vivo* competitive colonization model. *Journal of Bacteriology*.

[81] Pericone CD, Overweg K, Hermans PWM, Weiser JN (2000). Inhibitory and bactericidal effects of hydrogen peroxide production by *Streptococcus pneumoniae* on other inhabitants of the upper respiratory tract. *Infection and Immunity*.

[82] Rubins JB, Janoff EN (1998). Pneumolysin: a multifunctional pneumococcal virulence factor. *Journal of Laboratory and Clinical Medicine*.

[83] Jedrzejas MJ (2001). Pneumococcal virulence factors: structure and function. *Microbiology and Molecular Biology Reviews*.

[84] Mitchell AM, Mitchell TJ (2010). *Streptococcus pneumoniae*: virulence factors and variation. *Clinical Microbiology and Infection*.

[85] Marriott HM, Mitchell TJ, Dockrell DH (2008). Pneumolysin: a double-edged sword during the host-pathogen interaction. *Current Molecular Medicine*.

[86] Berry AM, Lock RA, Hansman D, Paton JC (1989). Contribution of autolysin to virulence of *Streptococcus pneumoniae*. *Infection and Immunity*.

[87] Mellroth P, Daniels R, Eberhardt A (2012). LytA, major autolysin of *Streptococcus pneumoniae*, requires access to nascent peptidoglycan. *Journal of Biological Chemistry*.

[88] Gosink KK, Mann ER, Guglielmo C, Tuomanen EI, Masure HR (2000). Role of novel choline binding proteins in virulence of *Streptococcus pneumoniae*. *Infection and Immunity*.

[89] Orihuela CJ, Mahdavi J, Thornton J (2009). Laminin receptor initiates bacterial contact with the blood brain barrier in experimental meningitis models. *Journal of Clinical Investigation*.

[90] Attali C, Frolet C, Durmort C, Offant J, Vernet T, Di Guilmi AM (2008). *Streptococcus pneumoniae* choline-binding protein E interaction with plasminogen/plasmin stimulates migration across the extracellular matrix. *Infection and Immunity*.

[91] Ren B, Szalai AJ, Thomas O, Hollingshead SK, Briles DE (2003). Both family 1 and family 2 PspA proteins can inhibit complement deposition and confer virulence to a capsular serotype 3 strain of *Streptococcus pneumoniae*. *Infection and Immunity*.

[92] Jedrzejas MJ (2006). Unveiling molecular mechanisms of pneumococcal surface protein A interactions with antibodies and lactoferrin. *Clinica Chimica Acta*.

[93] Voss S, Hallström T, Saleh M (2013). The choline-binding protein PspC of **Streptococcus pneumoniae** interacts with the C-terminal heparin-binding domain of vitronectin. *Journal of Biological Chemistry*.

[94] Holmes AR, McNab R, Millsap KW (2001). The pavA gene of *Streptococcus pneumoniae* encodes a fibronectin-binding protein that is essential for virulence. *Molecular Microbiology*.

[95] Pracht D, Elm C, Gerber J (2005). PavA of *Streptococcus pneumoniae* modulates adherence, invasion, and meningeal inflammation. *Infection and Immunity*.

[96] Jensch I, Gámez G, Rothe M (2010). PavB is a surface-exposed adhesin of *Streptococcus pneumoniae* contributing to nasopharyngeal colonization and airways infections. *Molecular Microbiology*.

[97] Anderton JM, Rajam G, Romero-Steiner S (2007). E-cadherin is a receptor for the common protein pneumococcal surface adhesin A (PsaA) of *Streptococcus pneumoniae*. *Microbial Pathogenesis*.

[98] Rajam G, Phillips DJ, White E (2008). A functional epitope of the pneumococcal surface adhesin A activates nasopharyngeal cells and increases bacterial internalization. *Microbial Pathogenesis*.

[99] Yamaguchi M, Terao Y, Mori Y, Hamada S, Kawabata S (2008). PfbA, a novel plasmin- and fibronectin-binding protein of *Streptococcus pneumoniae*, contributes to fibronectin-dependent adhesion and antiphagocytosis. *Journal of Biological Chemistry*.

[100] Dockrell DH, White MKB, Mitchell TJ (2012). Pneumococcal pneumonia: mechanisms of infection and resolution. *Chest*.

[101] Sanchez CJ, Kumar N, Lizcano A (2011). *Streptococcus pneumoniae* in biofilms are unable to cause invasive disease due to altered virulence determinant production. *PLoS ONE*.

[102] Zhang Y, Masi AW, Barniak V, Mountzouros K, Hostetter MK, Green BA (2001). Recombinant PhpA protein, a unique histidine motif-containing protein from *Streptococcus pneumoniae*, protects mice against intranasal pneumococcal challenge. *Infection and Immunity*.

[103] Trappetti C, Kadioglu A, Carter M (2009). Sialic acid: a preventable signal for pneumococcal biofilm formation, colonization, and invasion of the host. *Journal of Infectious Diseases*.

[104] Xu G, Potter JA, Russell RJM, Oggioni MR, Andrew PW, Taylor GL (2008). Crystal structure of the NanB sialidase from *Streptococcus pneumoniae*. *Journal of Molecular Biology*.

[105] Britton JL, Buckeridge TJ, Finn A, Kadioglu A, Jenkinson HF (2012). Pneumococcal neuraminidase A: an essential upper airway colonization factor for **Streptococcus pneumoniae**. *Molecular Oral Microbiology*.

[106] Parker D, Soong G, Planet P, Brower J, Ratner AJ, Prince A (2009). The NanA neuraminidase of *Streptococcus pneumoniae* is involved in biofilm formation. *Infection and Immunity*.

[107] Beiter K, Wartha F, Albiger B, Normark S, Zychlinsky A, Henriques-Normark B (2006). An endonuclease allows *Streptococcus pneumoniae* to escape from neutrophil extracellular traps. *Current Biology*.

[108] Moon AF, Midon M, Meiss G, Pingoud A, London RE, Pedersen LC (2011). Structural insights into catalytic and substrate binding mechanisms of the strategic EndA nuclease from *Streptococcus pneumoniae*. *Nucleic Acids Research*.

[109] Blue CE, Paterson GK, Kerr AR, Bergé M, Claverys JP, Mitchell TJ (2003). ZmpB, a novel virulence factor of *Streptococcus pneumoniae* that induces tumor necrosis factor alpha production in the respiratory tract. *Infection and Immunity*.

[110] Bergé M, García P, Iannelli F (2001). The puzzle of zmpB and extensive chain formation, autolysis defect and non-translocation of choline-binding proteins in *Streptococcus pneumoniae*. *Molecular Microbiology*.

[111] Niu S, Luo M, Tang J (2013). Structural basis of the novel *S. pneumoniae* virulence factor, GHIP, a glycosyl hydrolase 25 participating in host-cell invasion. *PLoS ONE*.

[112] Lee JO, Kim JY, Rhee DK, Pyo S (2013). *Streptococcus pneumoniae* ClpP protease induces apoptosis via caspase-dependent pathway in human neuroblastoma cells: cytoplasmic relocalization of p53. *Toxicon*.

[113] Beilharz K, Nováková L, Fadda D, Branny P, Massidda O, Veening J-W (2012). Control of cell division in *Streptococcus pneumoniae* by the conserved Ser/Thr protein kinase StkP. *Proceedings of the National Academy of Sciences of the United States of America*.

[114] Koppe U, Suttorp N, Opitz B (2012). Recognition of *Streptococcus pneumoniae* by the innate immune system. *Cellular Microbiology*.

[115] Saïd-Sadier N, Ojcius DM (2012). Alarmins, inflammasomes and immunity. *Biomedical Journal*.

[116] Malley R (2010). Antibody and cell-mediated immunity to *Streptococcus pneumoniae*: implications for vaccine development. *Journal of Molecular Medicine*.

[117] Olliver M, Hiew J, Mellroth P, Henriques-Normark B, Bergman P (2011). Human monocytes promote Th1 and Th17 responses to *Streptococcus pneumoniae*. *Infection and Immunity*.

[118] Weiser JN, Austrian R, Sreenivasan PK, Masure HR (1994). Phase variation in pneumococcal opacity: relationship between colonial morphology and nasopharyngeal colonization. *Infection and Immunity*.

[119] Henriques-Normark B, Tuomanen EI (2013). The pneumococcus: epidemiology, microbiology, and pathogenesis. *Cold Spring Harbour Perspectives in Medicine*.

[120] Hyams C, Opel S, Hanage W (2011). Effects of *Streptococcus pneumoniae* strain background on complement resistance. *PLoS ONE*.

[121] Hyams C, Trzcinski K, Camberlein E (2013). *Streptococcus pneumoniae* capsular serotype invasiveness correlates with the degree of factor H binding and opsonisation with C3b/iC3b. *Infection and Immunity*.

[122] Ogunniyi AD, Giammarinaro P, Paton JC (2002). The genes encoding virulence-associated proteins and the capsule of *Streptococcus pneumoniae* are upregulated and differentially expressed *in vivo*. *Microbiology*.

[123] Hammerschmidt S, Wolff S, Hocke A, Rosseau S, Müller E, Rohde M (2005). Illustration of pneumococcal polysaccharide capsule during adherence and invasion of epithelial cells. *Infection and Immunity*.

[124] Rodriguez JL, Dalia AB, Weiser JN (2012). Increased chain length promotes pneumococcal adherence and colonization. *Infection and Immunity*.

[125] Lizcano A, Chin T, Sauer K, Tuomanen EI, Orihuela CJ (2010). Early biofilm formation on microtiter plates is not correlated with the invasive disease potential of *Streptococcus pneumoniae*. *Microbial Pathogenesis*.

[126] Qin L, Kida Y, Imamura Y, Kuwano K, Watanabe H (2013). Impaired capsular polysaccharide is relevant to enhanced biofilm formation and lower virulence in *Streptococcus pneumoniae*. *Journal of Infection and Chemotherapy*.

[127] Mutepe ND, Cockeran R, Steel HC (2013). Effects of cigarette smoke condensate on pneumococcal biofilm formation and pneumolysin. *European Respiratory Journal*.

[128] Yesilkaya H, Andisi VF, Andrew PW, Bijlsma JJ (2013). *Streptococcus pneumoniae* and reactive oxygen species: an unusual approach to living with radicals. *Trends in Immunology*.

[129] Carvalho SM, Farshchi Andisi V, Gradstedt H (2013). Pyruvate oxidase influences the sugar utilization pattern and capsule production in *Streptococcus pneumoniae*. *PLoS ONE*.

[130] López R, García E (2004). Recent trends on the molecular biology of pneumococcal capsules, lytic enzymes, and bacteriophage. *FEMS Microbiology Reviews*.

[131] Xu G, Kiefel MJ, Wilson JC, Andrew PW, Oggioni MR, Taylor GL (2011). Three *Streptococcus pneumoniae* sialidases: three different products. *Journal of the American Chemical Society*.

[132] Krivan HC, Roberts DD, Ginsburg V (1988). Many pulmonary pathogenic bacteria bind specifically to the carbohydrate sequence GalNAc*β*1-4Gal found in some glycolipids. *Proceedings of the National Academy of Sciences of the United States of America*.

[133] Gualdi L, Hayre JK, Gerlini A (2012). Regulation of neuraminidase expression in *Streptococcus pneumoniae*. *BMC Microbiology*.

[134] Feldman C, Cockeran R, Jedrzejas MJ, Mitchell TJ, Anderson R (2007). Hyaluronidase augments pneumolysin-mediated injury to human ciliated epithelium. *International Journal of Infectious Diseases*.

[135] Littmann M, Albiger B, Frentzen A, Normark S, Henriques-Normark B, Plant L (2009). *Streptococcus pneumoniae* evades human dendritic cell surveillance by pneumolysin expression. *EMBO Molecular Medicine*.

[136] Witzenrath M, Gutbier B, Hocke AC (2006). Role of pneumolysin for the development of acute lung injury in pneumococcal pneumonia. *Critical Care Medicine*.

[137] McNeela EA, Burke Á, Neill DR (2010). Pneumolysin activates the NLRP3 inflammasome and promotes proinflammatory cytokines independently of TLR4. *PLoS Pathogens*.

[138] Cockeran R, Theron AJ, Steel HC (2001). Proinflammatory interactions of pneumolysin with human neutrophils. *Journal of Infectious Diseases*.

[139] Cockeran R, Steel HC, Mitchell TJ, Feldman C, Anderson R (2001). Pneumolysin potentiates production of prostaglandin E2 and leukotriene B4 by human neutrophils. *Infection and Immunity*.

[140] Fickl H, Cockeran R, Steel HC (2005). Pneumolysin-mediated activation of NF*κ*B in human neutrophils is antagonized by docosahexaenoic acid. *Clinical and Experimental Immunology*.

[141] Ratner AJ, Hippe KR, Aguilar JL, Bender MH, Nelson AL, Weiser JN (2006). Epithelial cells are sensitive detectors of bacterial pore-forming toxins. *Journal of Biological Chemistry*.

[142] Koga T, Jae HL, Jono H (2008). Tumor suppressor cylindromatosis acts as a negative regulator for *Streptococcus pneumoniae*-induced NFAT signaling. *Journal of Biological Chemistry*.

[143] Aguilar JL, Kulkarni R, Randis TM (2009). Phosphatase-dependent regulation of epithelial mitogen-activated protein kinase responses to toxin-induced membrane pores. *PLoS ONE*.

[144] Cockeran R, Durandt C, Feldman C, Mitchell TJ, Anderson R (2002). Pneumolysin activates the synthesis and release of interleukin-8 by human neutrophils in vitro. *Journal of Infectious Diseases*.

[145] Malley R, Henneke P, Morse SC (2003). Recognition of pneumolysin by Toll-like receptor 4 confers resistance to pneumococcal infection. *Proceedings of the National Academy of Sciences of the United States of America*.

[146] Barber GN (2011). Cytoplasmic DNA innate immune pathways. *Immunological Reviews*.

[147] Sharma S, Fitzgerald KA (2011). Innate immune sensing of DNA. *PLoS Pathogens*.

[148] Hahn S, Giaglis S, Chowdury CS, Hösli I, Hasler P (2013). Modulation of neutrophil NETosis: interplay between infectious agents and underlying host physiology. *Seminars in Immunopathology*.

[149] Mori Y, Yamaguchi M, Terao Y, Hamada S, Ooshima T, Kawabata S (2012). *α*-enolase of *Streptococcus pneumoniae* induces formation of neutrophil extracellular traps. *Journal of Biological Chemistry*.

[150] Moorthy AN, Narasaraju T, Rai P (2013). *In Vivo* and *in vitro* studies on the roles of neutrophil extracellular traps during secondary pneumococcal pneumonia after primary pulmonary influenza infection. *Frontiers in Immunology*.

[151] Saffarzadeh M, Juenemann C, Queisser MA (2012). Neutrophil extracellular traps directly induce epithelial and endothelial cell death: a predominant role of histones. *PLoS ONE*.

[152] Gough DR, Cotter TG (2011). Hydrogen peroxide: a Jekyll and Hyde signalling molecule. *Cell Death and Disease*.

[153] Veal E, Day A (2011). Hydrogen peroxide as a signaling molecule. *Antioxidants and Redox Signaling*.

[154] Anderson R, Steel HC, Cockeran R (2007). Comparison of the effects of macrolides, amoxicillin, ceftriaxone, doxycycline, tobramycin and fluoroquinolones, on the production of pneumolysin by *Streptococcus pneumoniae in vitro*. *Journal of Antimicrobial Chemotherapy*.

[155] Vanaudenaerde BM, Wuyts WA, Geudens N (2007). Macrolides inhibit IL17-induced IL8 and 8-isoprostane release from human airway smooth muscle cells. *The American Journal of Transplantation*.

[156] Kikuchi T, Hagiwara K, Honda Y (2002). Clarithromycin suppresses lipopolysaccharide-induced interleukin-8 production by human monocytes through AP-1 and NF-*Κ*B transcription factors. *Journal of Antimicrobial Chemotherapy*.

[157] Suzaki I, Asano K, Kanei A, Suzaki H (2013). Enhancement of thioredoxin production from nasal epithelial cells by the macrolide antibiotic, clarithromycin *in vitro*. *In Vivo*.

[158] Li M, Zhong X, He Z (2012). Effect of erythromycin on cigarette-induced histone deacetylase protein expression and nuclear factor-*κ*B activity in human macrophages *in vitro*. *International Immunopharmacology*.

[159] Giamarellos-Bourboulis EJ, Pechère J-C, Routsi C (2008). Effect of clarithromycin in patients with sepsis and ventilator-associated pneumonia. *Clinical Infectious Diseases*.

[160] Walkey AJ, Wiener RS (2012). Macrolide antibiotics and survival in patients with acute lung injury. *Chest*.

[161] Karlström Å, Boyd KL, English BK, McCullers JA (2009). Treatment with protein synthesis inhibitors improves outcomes of secondary bacterial pneumonia after influenza. *Journal of Infectious Diseases*.

[162] Wunderink RG (2009). Adjunctive therapy in community-acquired pneumonia. *Seminars in Respiratory and Critical Care Medicine*.

[163] Noto MJ, Wheeler AP (2012). Macrolides for acute lung injury. *Chest*.

[164] Barnes PJ (2007). New molecular targets for the treatment of neutrophilic diseases. *Journal of Allergy and Clinical Immunology*.

[165] Brouwer MC, Heckenberg SGB, de Gans J, Spanjaard L, Reitsma JB, van de Beek D (2010). Nationwide implementation of adjunctive dexamethasone therapy for pneumococcal meningitis. *Neurology*.

[166] Confalonieri M, Urbino R, Potena A (2005). Hydrocortisone infusion for severe community-acquired pneumonia: a preliminary randomized study. *The American Journal of Respiratory and Critical Care Medicine*.

[167] Garcia-Vidal C, Calbo E, Pascual V, Ferrer C, Quintana S, Garau J (2007). Effects of systemic steroids in patients with severe community-acquired pneumonia. *European Respiratory Journal*.

[168] Mikami K, Suzuki M, Kitagawa H (2007). Efficacy of corticosteroids in the treatment of community-acquired pneumonia requiring hospitalization. *Lung*.

[169] Snijders D, Daniels JMA, de Graaff CS, van der Werf TS, Boersma WG (2010). Efficacy of corticosteroids in community-acquired pneumonia: a randomized double-blinded clinical trial. *The American Journal of Respiratory and Critical Care Medicine*.

[170] Polverino E, Cillóniz C, Dambrava P (2013). Systemic corticosteroids for community-acquired pneumonia: reasons for use and lack of benefit on outcome. *Respirology*.

[171] Meijvis SCA, Hardeman H, Remmelts HHF (2011). Dexamethasone and length of hospital stay in patients with community-acquired pneumonia: a randomised, double-blind, placebo-controlled trial. *The Lancet*.

[172] Remmelts HH, Meijvis SCA, Biesma DH (2012). Dexamethasone downregulates the systemic cytokine response in patients with community-acquired pneumonia. *Clinical and Vaccine Immunology*.

[173] Nie W, Zhang Y, Cheng J, Xiu Q (2012). Corticosteroids in the treatment of community-acquired pneumonia in adults: a meta-analysis. *PLoS ONE*.

[174] Extended Steroid in CAP(e) (ESCAPe). http://clinicaltrials.gov/show/NCT01283009.

[175] Mira E, Mañes S (2009). Immunomodulatory and anti-inflammatory activities of statins. *Endocrine, Metabolic and Immune Disorders*.

[176] Massaro M, Zampolli A, Scoditti E (2010). Statins inhibit cyclooxygenase-2 and matrix metalloproteinase-9 in human endothelial cells: anti-angiogenic actions possibly contributing to plaque stability. *Cardiovascular Research*.

[177] Wang Y, Zhang MX, Meng X (2011). Atorvastatin suppresses LPS-induced rapid upregulation of toll-like receptor 4 and its signaling pathway in endothelial cells. *The American Journal of Physiology*.

[178] Leung P-O, Wang S-H, Lu S-H, Chou W-H, Shiau C-Y, Chou T-C (2011). Simvastatin inhibits pro-inflammatory mediators through induction of heme oxygenase-1 expression in lipopolysaccharide-stimulated RAW264.7 macrophages. *Toxicology Letters*.

[179] Mrad MF, Mouawad CA, Al-Hariri M, Eid AA, Alam J, Habib A (2012). Statins modulate transcriptional activity of heme-oxygenase-1 promoter in NIH 3T3 cells. *Journal of Cellular Biochemistry*.

[180] Serezani CH, Ballinger MN, Aronoff DM, Peters-Golden M (2008). Cyclic AMP: master regulator of innate immune cell function. *The American Journal of Respiratory Cell and Molecular Biology*.

[181] Matthay MA, Brower RG, Carson S (2011). Randomized, placebo-controlled clinical trial of an aerosolized *β*2-agonist for treatment of acute lung injury. *The American Journal of Respiratory and Critical Care Medicine*.

[182] Smith FG, Perkins GD, Gates S (2012). Effect of intravenous *β*-2 agonist treatment on clinical outcomes in acute respiratory distress syndrome (BALTI-2): a multicentre, randomised controlled trial. *The Lancet*.

[183] Gravett CM, Theron AJ, Steel HC (2010). Interactive inhibitory effects of formoterol and montelukast on activated human neutrophils. *European Respiratory Journal*.

[184] Stables MJ, Newson J, Ayoub SS, Brown J, Hyams CJ, Gilroy DW (2010). Priming innate immune responses to infection by cyclooxygenase inhibition kills antibiotic-susceptible and -resistant bacteria. *Blood*.

